# The Soluble Guanylate Cyclase Stimulator Riociguat Ameliorates Pulmonary Hypertension Induced by Hypoxia and SU5416 in Rats

**DOI:** 10.1371/journal.pone.0043433

**Published:** 2012-08-17

**Authors:** Michaela Lang, Baktybek Kojonazarov, Xia Tian, Anuar Kalymbetov, Norbert Weissmann, Friedrich Grimminger, Axel Kretschmer, Johannes-Peter Stasch, Werner Seeger, Hossein Ardeschir Ghofrani, Ralph Theo Schermuly

**Affiliations:** 1 University of Giessen and Marburg Lung Center, Giessen, Germany; 2 Bayer HealthCare, Wuppertal, Germany; 3 Department of Lung Development and Remodeling, Max Planck Institute for Heart and Lung Research, Bad Nauheim, Germany; Virginia Commonwealth University, United States of America

## Abstract

**Background:**

The nitric oxide (NO)–soluble guanylate cyclase (sGC)–cyclic guanosine monophosphate (cGMP) signal-transduction pathway is impaired in many cardiovascular diseases, including pulmonary arterial hypertension (PAH). Riociguat (BAY 63–2521) is a stimulator of sGC that works both in synergy with and independently of NO to increase levels of cGMP. The aims of this study were to investigate the role of NO–sGC–cGMP signaling in a model of severe PAH and to evaluate the effects of sGC stimulation by riociguat and PDE5 inhibition by sildenafil on pulmonary hemodynamics and vascular remodeling in severe experimental PAH.

**Methods and Results:**

Severe angioproliferative PAH was induced in rats by combined exposure to the vascular endothelial growth factor receptor antagonist SU5416 and hypoxia (SUHx). Twenty-one days thereafter rats were randomized to receive either riociguat (10 mg/kg/day), sildenafil (50 mg/kg/day) or vehicle by oral gavage, for 14 days until the day of the terminal hemodynamic measurements. Administration of riociguat or sildenafil significantly decreased right ventricular systolic pressure (RVSP). Riociguat significantly decreased RV hypertrophy (RVH) (0.55±0.02, *p*<0.05), increased cardiac output (60.8±.8 mL/minute, *p*<0.05) and decreased total pulmonary resistance (4.03±0.3 mmHg min^−1^ ml^−1^ 100 g BW, *p*<0.05), compared with sildenafil and vehicle. Both compounds significantly decreased the RV collagen content and improved RV function, but the effects of riociguat on tricuspid annular plane systolic excursion and RV myocardial performance were significantly better than those of sildenafil (*p*<0.05). The proportion of occluded arteries was significantly lower in animals receiving riociguat than in those receiving vehicle (*p*<0.05); furthermore, the neointima/media ratio was significantly lower in those receiving riociguat than in those receiving sildenafil or vehicle (*p*<0.05).

**Conclusion:**

Riociguat and sildenafil significantly reduced RVSP and RVH, and improved RV function compared with vehicle. Riociguat had a greater effect on hemodynamics and RVH than sildenafil.

## Introduction

Pulmonary arterial hypertension (PAH) is a disease of the small pulmonary arteries, characterized by excessive pulmonary vascular remodeling leading to a progressive increase in pulmonary vascular resistance (PVR), progressive right ventricular hypertrophy (RVH), heart failure and death. Pathological changes in the pulmonary arteries of patients with PAH include smooth muscle and fibroblast proliferation, neointima formation, complex plexiform lesions and vascular obstruction [1].

The pathophysiological mechanisms of PAH are complex and multifactorial. Endothelial dysfunction has been strongly implicated as an initiating factor in the development of PAH [2]. The nitric oxide (NO)–soluble guanylate cyclase (sGC)–cyclic guanosine monophosphate (cGMP) signal-transduction pathway plays an important role in the regulation of pulmonary vascular tone and resistance in PAH [3].

Despite recent advances in the treatment of PAH, most of the available classes of drug, such as prostacyclin analogs, endothelin receptor antagonists and phosphodiesterase type 5 (PDE-5) inhibitors, have significant limitations. For example, while PDE-5 inhibitors prevent the degradation of cGMP and have demonstrated efficacy in PAH [1], [4], their effects are dependent on the presence of an intact NO–sGC–cGMP axis [5]. Hence, depletion of NO by reduced synthesis or increased NO scavenging can impair cGMP production and thereby limit the efficacy of these drugs [6].

It has been shown that direct pharmacological stimulation of sGC by BAY 41–2272 results in pulmonary vasodilation in lambs [7] and reverses hemodynamic and structural changes associated with monocrotaline- and hypoxia-induced pulmonary hypertension (PH) in rats and mice [8]. Riociguat (BAY 63–2521; Bayer Healthcare AG, Wuppertal, Germany) is a novel NO-independent stimulator of sGC that increases the sensitivity of sGC to endogenous bioavailable NO and mimics the effects described above when NO is absent or depleted [6]. Data from a recent phase 2 clinical study have shown that riociguat has a favorable safety profile and results in improved exercise capacity and pulmonary hemodynamics in patients with chronic thromboembolic PH and PAH [9].

Monocrotaline and hypoxic models are the most frequently used experimental models in the development of novel therapeutic strategies for PAH. However, these models do not produce the occlusive neointimal and plexiform lesions that are observed in human PAH [10], [11], [12]. Combined use of the vascular endothelial growth factor receptor (VEGFR) inhibitor SU5416 and exposure to hypoxia (SUHx) in rats leads to severe PAH and closely mimics the vascular changes seen in patients with severe PAH [13], [14], [15]. Although this model has been used by investigators to test new drug treatments, the reversibility of the plexiform lesions still remains questionable [16]. Additionally, the role of NO–sGC–cGMP in this model has not yet been studied.

Therefore, we set out to investigate the role of NO–sGC–cGMP signaling in the SUHx model of severe PAH and to evaluate the effects of sGC stimulation by riociguat and of PDE-5 inhibition by sildenafil on pulmonary hemodynamics and vascular remodeling in severe experimental PAH.

## Materials and Methods

All experiments were performed according to institutional guidelines complying with national and international regulations. Both the University Animal Care Committee and the Federal Authorities for Animal Research of the Regierungspräsidium Giessen (Hessen, Germany) approved the study protocols (GI 20/10 Nr. 03/2009 and GI 20/10 Nr. 80/2009).

**Table 1:** Baseline and post-treatment characteristics of the studied groups.

Characteristic	Healthy controls (*n* = 9)	SUHx animals						
		3-week controls (*n* = 7)	Vehicle (*n* = 9)		Sildenafil (*n* = 9)		Riociguat (*n* = 9)	
			Pre-treatment	Post-treatment	Pre-treatment	Post-treatment	Pre-treatment	Post-treatment
‘Notch’ duration, msec	-	42.00±0.93	43.65±1.2	63.3±1.6*	45.12±2.1	55.3±1.8^†§^	45.72±2.53	45.3±1.8^§^
PAAT, msec	36.56±0.65	18.21±0.89	18.52±0.86	12.19±0.73*	18.61±0.97	16.94±0.69^§^	18.33±0.89	19.38±1.39^§^
RVID, mm	2.76±0.07	3.97±0.06	3.9±0.09	4.3±0.4*	3.86±0.09	3.77±0.23^§^	3.95±0.02^†^	3.56±0.07^‡§¶^
RVWT, mm	0.51±0.01	1.30±0.02	1.30±0.05	1.64±0.07*	1.31±0.03	1.37±0.03^§^	1.33±0.03	1.27±0.04^‡¶^
TAPSE, mm	2.62±0.02	1.52±0.06	1.56±0.03	1.32±0.09*	1.50±0.06	1.78±0.06^†§^	1.53±0.03	2.18±0.04^‡¶^
MPI	0.63±0.06	1.85±0.08	1.82±0.05	2.44±0.16*	1.83±0.06	1.51±0.04^†§^	1.83±0.11	1.25±0.05^‡§¶^

* ***p*** **≤0.05 vs pre-treatment vehicle; ^†^** ***p*** **≤0.05 vs pre-treatment sildenafil; ^‡^** ***p*** **≤0.05 vs pre-treatment riociguat; ^§^** ***p*** **≤0.05 vs post-treatment vehicle; ^¶^** ***p*** **≤0.05 vs post-treatment sildenafil.**

***Note.*** **MPI, myocardial performance index; ‘notch’ duration, pulmonary artery flow mid-systolic notch duration; PAAT, pulmonary artery acceleration time; RVID, right ventricular internal diameter, RVWT, right ventricular wall thickness, TAPSE, tricuspid annular plane systolic excursion.**

### Animals

Adult male Sprague-Dawley rats with a body weight of 200–250 g were obtained from Charles River Laboratories (Wilmington, MA, USA). Rats were injected subcutaneously with the VEGFR inhibitor SU5416 (20 mg/kg) under isoflurane anesthesia and exposed to chronic hypoxia (10% oxygen) for 3 weeks (SUHx). Twenty-one days thereafter animals were treated orally by gavage with riociguat (BAY 63–2521; Bayer Healthcare AG, Wuppertal, Germany) at a dose of 10 mg/kg/day (*n* = 9), sildenafil (Pfizer Inc., New York, NY, USA) at a dose of 50 mg/kg/day (*n* = 9) or vehicle (*n* = 9) for 14 days until the day of the final hemodynamic measurements. Rats injected with saline and exposed to normoxic gas for 35 days were used as a healthy control group (*n* = 9). Additionally, one group of SUHx animals were used as 3-week controls (*n* = 7).

**Figure 1:**
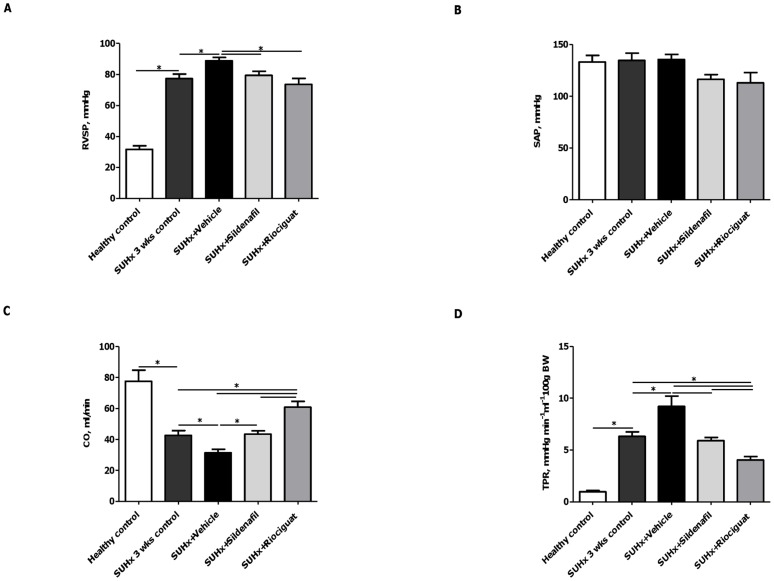
Effects of riociguat and sildenafil on hemodynamics in SUHx rats. (A) RVSP, (B) SAP, (C) CO and (D) TPR of different treatment groups. *p<0.05. *Note*. CO, cardiac output; RVSP, right ventricular systolic pressure; SAP, systemic arterial pressure; TPR, total pulmonary resistance.

**Figure 2:**
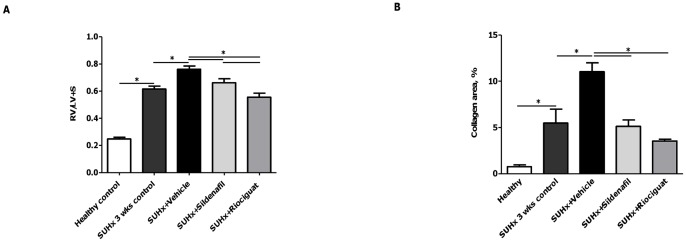
Effects of riociguat and sildenafil on RVH and fibrosis in SUHx rats. (A) Ratio of the RV to LV+S and (B) RV collagen contents of different treatment groups. *p<0.05. *Note.* LV, left ventricle; RV, right ventricle; RVH, right ventricular hypertrophy; S, interventricular septum.

### Hemodynamic measurements

Invasive hemodynamic measurements, including right ventricular systolic pressure (RVSP), cardiac output (CO), total pulmonary resistance (TPR) and systemic arterial pressure (SAP) were performed as described previously [17].

**Figure 3:**
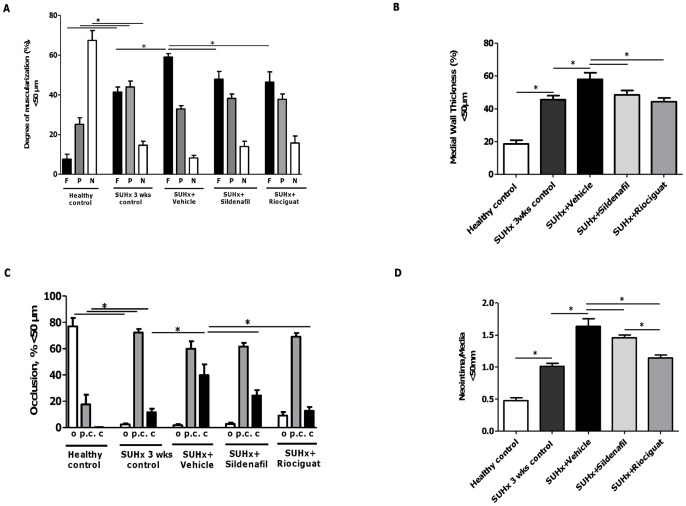
Effects of riociguat and sildenafil on pulmonary vascular remodeling in SUHx rats. (A) Proportion of non-, partially or fully muscularized pulmonary arteries as a percentage of the total pulmonary artery cross section (<50 µm); (B) proportion of medial wall thickness of the small pulmonary arteries (<50 µm); (C) proportion of opened, partly closed and closed small pulmonary arteries (<50 µm) and (D) neointima/media ratio of the small pulmonary arteries (<50 µm) of different treatment groups. *p<0.05. *Note.* c, closed; F, fully muscularized; N, non-muscularized; o, opened; P, partially muscularized; pc, partly closed.

### Assessment of RV hypertrophy

After excision of the heart, the right ventricular (RV) wall was separated from the left ventricular (LV) wall and the interventricular septum (S). The ratio of the RV to LV plus S weight (RV/[LV+S]) was calculated as an index of RVH.

**Figure 4:**
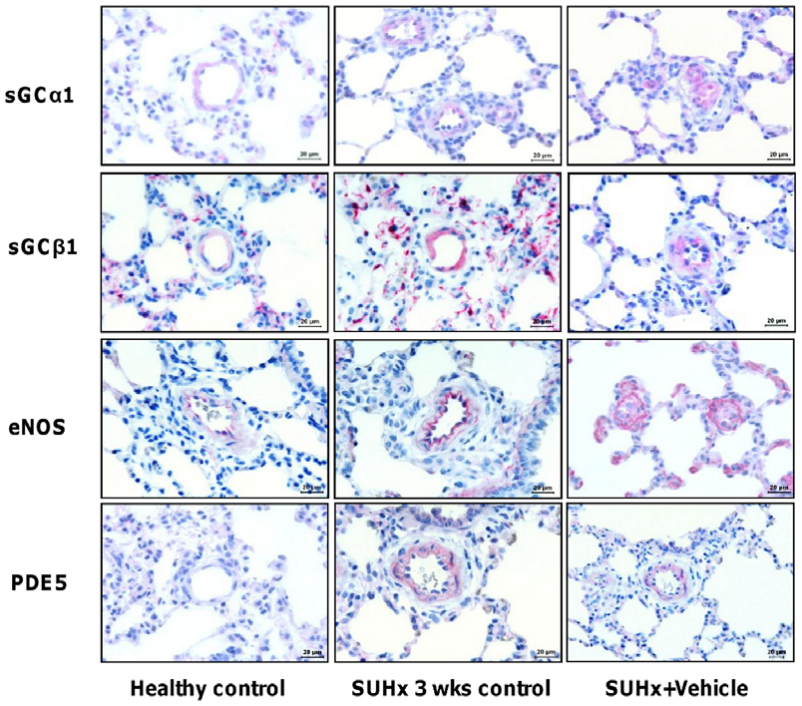
Localization of sGCα1, sGCβ1, eNOS and PDE-5 in pulmonary vessels of SUHx rats. Localization of sGCα1, sGCβ1, eNOS and PDE-5 are presented in healthy controls, SUHx 3-week control animals and vehicle-treated animals. *Note.* eNOS, endothelial nitric oxide synthase; PDE-5, phosphodiesterase type 5; sGCα1, soluble guanylate cyclase α1; sGCβ1, soluble guanylate cyclase β1.

### Echocardiography

Anesthesia was induced with 3% isoflurane gas and maintained with 1.0–1.5% isoflurane in room air supplemented with 100% oxygen. Transthoracic echocardiography was performed with a Vevo770 high-resolution imaging system equipped with a 25 MHz transducer (VisualSonics, Toronto, ON, Canada). Pulmonary artery acceleration time (PAAT), pulmonary artery flow mid-systolic ‘notch’ duration, RV internal diameter (RVID), right ventricular wall thickness (RVWT), tricuspid annular plane systolic excursion (TAPSE), and RV myocardial performance index (MPI), or Tei index, [18] were measured as described previously [19], [20].

**Figure 5:**
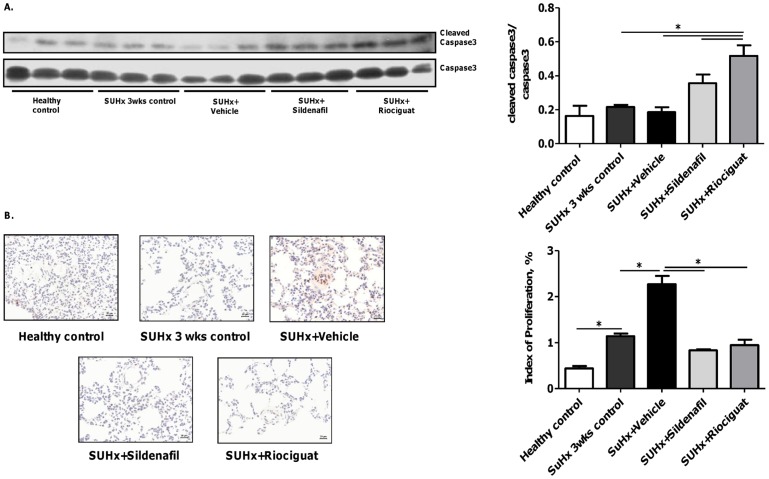
Effects of riociguat and sildenafil on apoptosis and cell proliferation in the lung tissue of SUHx rats. (A) Western blotting of caspase-3 and cleaved caspase-3 with densitometric analysis and (B) PCNA immunostaining with representative examples of different treatment groups. *p<0.05. *Note.* PCNA, proliferating cell nuclear antigen.

### Pulmonary vascular morphology

Lung tissue preparation, sectioning, staining with hematoxylin and pulmonary vascular morphometry were performed as described previously [15], [17]. The neointima/media ratio was calculated in 80–100 muscular arteries with an external diameter smaller than 50 µm. Additionally, an occlusion score was used, as described by Oka et al. [14].

**Figure 6:**
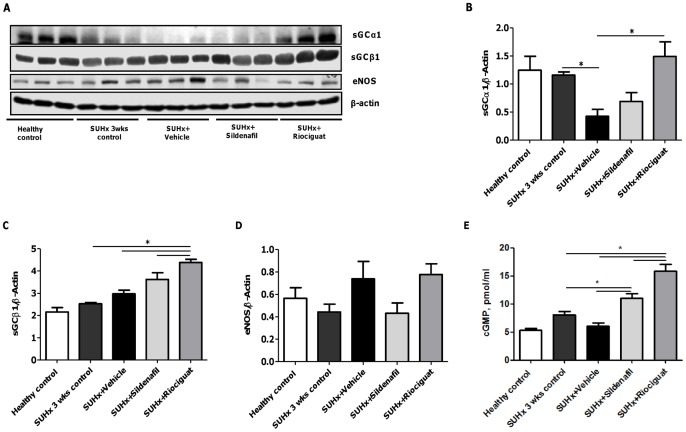
Effects of riociguat and sildenafil on sGC and eNOS protein levels, and cGMP concentration in the lung tissue of SUHx rats. (A) Western blotting of eNOS, sGCα1 and sGCβ1, (B) densitometric analysis of sGCα1/β-actin, (C) densitometric analysis of sGCβ1/β-actin, (D) densitometric analysis of eNOS/β-actin and (E) concentration of cGMP, in lung tissue of different treatment groups. *p<0.05. *Note.* eNOS, endothelial nitric oxide synthase; PDE-5, phosphodiesterase type 5; sGCα1, soluble guanylate cyclase α1; sGC, soluble guanylate cyclase; sGCβ1, soluble guanylate cyclase β1; cGMP, cyclic guanosine monophosphate.

### Immunohistochemistry and proliferating cell nuclear antigen

Paraffin-embedded lung tissue sectioned at 3 mm thickness was deparaffinized in xylene and rehydrated in a graded ethanol series to phosphate-buffered saline (pH 7.2). Antigen retrieval was performed by autoclaving in citrate buffer (pH 6.0) for 15 minutes. Immunohistochemical staining was performed using antibodies raised in rabbits against soluble guanylate cyclase α1 (sGCα1) (Sigma-Aldrich^®^, St Louis, MO, USA), soluble guanylate cyclase β1 (sGCβ1) (Abcam^®^, Cambridge, CA, USA), endothelial nitric oxide synthase (eNOS) (Enzo^®^ Life Sciences, Farmingdale, NY, USA) and PDE-5 (Cell Signaling Technology^®^, Danvers, MA, USA), as described previously [21]. Proliferating cell nuclear antigen (PCNA) staining was performed with rabbit polyclonal anti-PCNA antibody (Santa Cruz Biotechnology^®^ Inc., Santa Cruz, CA, USA), as described previously [22], [23].

### Western blot analysis

Protein from lung tissue was extracted by radioimmunoprecipitation assay (RIPA) buffer (Santa Cruz Biotechnology Inc., Santa Cruz, CA, USA). Denatured proteins were resolved by sodium dodecyl sulfate polyacrylamide gel electrophoresis (10% acrylamide) and transferred onto nitrocellulose membranes. After being blocked with 5% non-fat milk for 1 hour at room temperature, membranes were probed with primary antibodies overnight at 4°C (rabbit anti-sGCα1 antibody (Sigma-Aldrich, St Louis, MO, USA); rabbit anti-sGCβ1 antibody (Cayman Chemical, Ann Arbor, MI, USA); mouse anti-eNOS antibody (Enzo Life Sciences, Farmingdale, NY, USA); rabbit anti-caspase-3 antibody, rabbit anti-cleaved caspase-3 antibody (Cell Signaling Technology, Danvers, MA, USA); mouse anti-β-actin antibody (Abcam^®^, Cambridge, CA, USA). Following washing with tris-buffered saline containing 0.1% Triton X-100, horseradish peroxidase-conjugated secondary antibodies (anti-rabbit and anti-mouse, Sigma-Aldrich, St Louis, MO, USA) were applied for 1 hour. After washing, the blots were developed with an enhanced chemiluminescence kit (Amersham Bioscience, Piscataway, NJ, USA) followed by film exposure. The expression of protein was quantified by densitometry as the ratio of target protein divided by β-actin.

### cGMP measurement by enzyme immunoassay

cGMP levels in lung tissue were determined by the cGMP enzyme immunoassay (EIA) (Cayman Chemical, Ann Arbor, MI, USA) according to the manufacturer's instructions. A 50 mg sample of frozen lung tissue was homogenized in 0.5 mL 5% trichloroacetic acid (TCA). The supernatant was subjected to TCA extraction using 2.5 mL water-saturated ether three times and the residual ether from the aqueous layer was removed by heating the sample to 70°C. After centrifugation, the supernatants were normalized to the same protein concentration for use. Next, 50 μL of five-times diluted samples and standard solutions were incubated with 50 μL of tracer and 50 μL of antibody at 4°C overnight. After washing five times, plates were incubated with Ellman's solution for 120 minutes at room temperature with gentle shaking. The plates were read at a wavelength of 420 nm, and the standard curve was produced using the Cayman EIATriple workbook (Cayman Chemical, Ann Arbor, MI, USA). The sample cGMP concentration was determined (as pmol/mg tissue) using the equation obtained from the standard curve. Each sample was determined in triplicate and the process repeated twice.

### RV collagen content assay

RV was fixed in 4% formalin and stained using picrosirius red to determine interstitial collagen fractions, as described previously [19].

### Data analysis

All data are given as mean ± standard error of the mean (SEM). The different groups were compared using one-way analysis of variance (ANOVA) and subsequent application of the Newman–Keuls test. The differences between pre- and post-treatment echocardiographic values were assessed by two-way ANOVA with Bonferroni correction. Comparisons were considered statistically significant at *p*<0.05. Correlation between occluded vessel density and RVSP was assessed by linear regression analysis.

## Results

SUHx animals were randomized into the four groups (Table 1). Noninvasive evaluation of RV function demonstrated that SUHx rats developed severe RVH and RV failure, which progressively worsened from day 21 to day 35.

### Effects of active treatments on hemodynamics

Sildenafil and riociguat significantly reduced RVSP to 73.7±3.8 mmHg and 79.4±2.7 mmHg, respectively (*p*<0.05 versus vehicle) (Fig. 1A). Importantly, no changes in SAP (Fig. 1B) and heart rate (data not shown) were noted after sildenafil or riociguat administration. Additionally, pulmonary artery flow mid-systolic ‘notch’ duration and PAAT, measured by echocardiography, significantly improved in rats receiving sildenafil or riociguat, compared with those treated with vehicle, with values being comparable to the SUHx 3-week control animals (Table 1)**.** Both compounds markedly increased CO and in parallel significantly decreased TPR, compared with vehicle (Fig. 1C and Fig. 1D), with riociguat having a significantly greater effect than sildenafil. Riociguat also achieved a significantly greater increase in CO and decrease in TPR when compared with the SUHx 3-week controls (Fig. 1C and Fig. 1D).

### Effects of active treatments on RVH, RV function and RV fibrosis

Both study drugs significantly decreased RVH and RV fibrosis, and markedly improved RV function, as measured by echocardiography. Therefore, the RV/[LV+S] ratio (Fig. 2A) and right ventricular wall thickness (Table 1) were significantly lower in the riociguat and sildenafil groups (both *p*<0.05) than in the vehicle group, and were comparable with values in the SUHx 3-week control animals. Additionally, both riociguat and sildenafil significantly reduced RV dilatation and improved RV function, measured by M-mode (TAPSE) and tissue Doppler imaging (MPI), respectively, compared with both the SUHx 3-week control animals (*p*<0.05) and vehicle group (*p*<0.05) (Table 1). Notably, the effects of riociguat on TAPSE and MPI were significantly greater than those of sildenafil (*p*<0.05). In parallel, both compounds significantly reduced RV collagen content compared with vehicle (both *p*<0.05, Fig. 2B, Fig. S1).

### Effects of active treatments on pulmonary vascular remodeling

VEGFR blockade by SU5416 and exposure to hypoxia for 3 weeks caused severe PAH with development of occlusive lesions (Fig. 3A–C). Both compounds prevented further development of occlusive lesions as compared to vehicle, however, there was no complete reversal of the histological changes of the SUHx model.

The proportions of fully muscularized arteries were significantly lower in riociguat- and sildenafil-treated animals than in the vehicle group (both *p*<0.01), and were comparable with the SUHx 3-week control animals (Fig. 3A). Likewise, medial wall thickness was significantly lower in riociguat- and sildenafil-treated animals than in the vehicle group (*p*<0.05), but not compared with the SUHx 3-week control animals (Fig. 3B). The proportion of fully closed arteries was significantly lower after riociguat and sildenafil administration than after vehicle administration (*p*<0.05), while the proportion of opened arteries was not significantly different among the studied groups (Fig. 3C). Riociguat treatment was also associated with a significantly lower neointima/media ratio than sildenafil (*p*<0.05) or vehicle (*p*<0.05) (Fig. 3D).

### Immunohistochemistry

Immunohistochemical staining for eNOS, sGCα1, sGCβ1 and PDE-5 proteins is presented in Figure 4. Interestingly, immunohistochemistry demonstrated expression of sGCα1 and sGCβ1 in the medial wall of pulmonary arteries in SUHx 3 weeks control animals and in both the medial wall and endothelial cells 5 weeks after hypoxic exposure. eNOS was expressed in endothelial cells, while PDE-5 was extensively expressed in smooth muscle cells.

### Effects of active treatment on cleaved caspase-3 and pulmonary vascular cell proliferation

The Western blot of the lung tissue showed increased activation of caspase-3 in response to riociguat treatment compared with 3-week control animals and vehicle-treated animals (both *p*<0.05; Fig. 5A). Sildenafil also appeared to induce activation of caspase-3, but this effect was not significant. Additionally, the effects of sildenafil and riociguat on cell proliferation were analyzed by immunostaining for PCNA. We observed that immunoreactivity for PCNA was significantly increased in the lungs from all groups of SUHx animals compared with healthy controls (Fig. 5B). The index of proliferation was significantly reduced in the rats that received sildenafil or riociguat compared with vehicle-treated rats (both *p*<0.05).

### Effects of active treatment on sGC and eNOS lung protein levels

Levels of sGCα1 protein expression decreased in the SUHx 3-week control animals and were further reduced in vehicle-treated rats, while sGCβ1 levels were not affected by SUHx (Fig. 6A–C). Treatment with riociguat, but not sildenafil, was associated with increased expression of both sGCα1 and sGCβ1 in lung homogenate, compared with vehicle (*p*<0.05). eNOS levels were lower in the SUHx 3-week control animals and sildenafil-treated animals than in healthy controls, but increased after riociguat treatment; however, these changes were not significant (Fig. 6D).

### Effects of active treatment on cGMP levels

The levels of cGMP were not significantly affected by SUHx (Fig. 6E). Sildenafil and riociguat both induced a marked increase in cGMP levels compared with the SUHx 3-week control animals (*p*<0.05) and vehicle-treated animals (*p*<0.05).

## Discussion

Our study demonstrated that: (1) a combination of SU5416 and chronic exposure to hypoxic conditions resulted in severe PAH, RVH and RV failure, with evidence of severe changes in pulmonary vasculature, which closely mimicked the vascular changes seen in patients with severe PAH, as described by other investigators [13], [14], [15]; (2) the reduction in RVSP and RVH by riociguat and sildenafil was statistically significant, and RV function was also improved compared with vehicle, with the effects of riociguat on hemodynamics and RVH being greater than those of sildenafil; (3) riociguat prevented further progression of pulmonary vascular remodeling and formation of occlusive lesions in SUHx-exposed rats; (4) riociguat induced apoptosis and inhibited proliferation of pulmonary artery cells; and (5) treatment with riociguat led to significant up-regulation of sGCα and sGCβ expression, and increased cGMP production.

We have successfully established the SUHx model of PAH in our laboratory, having demonstrated that SUHx leads to the development of severe changes in pulmonary vasculature that closely mimic the vascular changes seen in patients with severe PAH. Additionally, we have confirmed the linear relationship between the proportions of occluded vessels and the RVSP (Fig. S2), as previously described by Oka et al. [14].

The NO–sGC–cGMP signaling pathway plays a crucial role in PH. The reduced bioavailability of NO or insensitivity of sGC to NO, due to oxidation or loss of the heme group, leads to decreased cGMP production and PH [24], [25], [26]. Many studies have demonstrated the role of NO in hypoxic pulmonary vasoconstriction and PH, but the role of NO signaling in the SUHx model of PH had still to be elucidated. In our study, we found that expression of sGCα was significantly decreased in SUHx rats treated with vehicle, while the expression of eNOS and sGCβ was not significantly different from that in healthy controls. Immunohistochemistry revealed that PDE-5 expression was also increased in SUHx rats. Interestingly, cGMP levels in the lung tissue of SUHx 3-week control animals were also comparable to levels observed in healthy controls.

We have previously demonstrated that levels of sGC subunits did not change significantly in hypoxic mice, but were significantly down-regulated in eNOS-deficient mice. Treatment by an sGC activator and stimulator significantly increased sGCβ, and reduced RVSP and pulmonary vascular remodeling in wild type but not in eNOS-deficient mice [8]. This suggests that the effects of sGC stimulators and activators depend on ongoing eNOS-dependent NO generation. However, in other studies, up-regulation of sGC has been reported in rats and mice exposed to hypoxia [21], [27], [28]. Additionally, it has been shown that PDE-5 activity is increased in animal models of PAH and human pulmonary artery cells [29], [30], [31]. However, the therapeutic effect of PDE-5 inhibitors is dependent on baseline NO expression (levels of which are typically reduced in PAH) and they are not effective in all patients [32], [33] and sometimes need to be given at doses above those licensed in order to be effective [1]. Taking into account all of these findings, we investigated whether the additional stimulation of sGC by riociguat or inhibition of PDE-5 by sildenafil improved pulmonary vascular hemodynamics and remodeling in SUHx rats.

Riociguat is a first-in-class drug that augments cGMP biosynthesis, promoting vasodilatation by direct stimulation of sGC in an NO-independent fashion, and by sensitization of sGC to low endogenous NO levels [34]. Recently, our group demonstrated that riociguat reverses RVSP, RVH and structural changes provoked by hypoxia in mice and by monocrotaline in rats [21], but not by combined VEGFR blockade and hypoxia in rats (SUHx).

We found that the stimulation of sGC by riociguat led to a more than twofold increase in the expression of both sGCα1 and sGCβ1 in the lungs of SUHx rats compared with vehicle and 3-week control animals. Both sildenafil and riociguat markedly increased cGMP levels in the lung tissue of SUHx rats, but the effect of riociguat was more pronounced. Thus, we have discovered that stimulation of sGC by riociguat leads to up-regulation of sGC expression and increased cGMP levels in hypoxic lungs, which is accompanied by improved hemodynamics in severe angioproliferative PH (SUHx).

In our study, we demonstrated that sildenafil and riociguat significantly reduced RVSP and pulmonary artery flow mid-systolic ‘notch’ duration compared with vehicle-treated animals. PAAT, a parameter that is strongly correlated with mean pulmonary arterial pressure as well as RVSP in humans and animals [19], [20], [35], significantly improved in response to sildenafil and riociguat treatment by 28% and 38%, respectively, compared with vehicle. Moreover, TPR, which is considered mainly to reflect the functional status of the pulmonary vascular endothelium/smooth-muscle-cell-coupled system and to provide important information when assessing the response to available treatments [36], was significantly reduced in SUHx rats treated with sildenafil or riociguat. Interestingly, in our study, the effects of riociguat on hemodynamics were comparable with results observed in a recently published phase 2 study [9], [37].

All of these positive changes in hemodynamics were accompanied by significant decreases in the proportion of fully muscularized arteries and suppression of medial wall thickening in both treated groups compared with vehicle, but not compared with the SUHx 3-week control animals. Importantly, sildenafil and riociguat prevented further development of occlusive lesions in SUHx rats. Notably, the effects of riociguat on pulmonary hemodynamics and vascular remodeling were superior to those of sildenafil. Importantly, riociguat increased cleaved caspase-3 and reduced numbers of PCNA-positive pulmonary artery cells. The effects of sildenafil were more strongly associated with reduced pulmonary artery cell proliferation than with activation of caspase-3, which is in line with results from previously published studies in MCT PH and in isolated human pulmonary artery cells [38], [31].

The cardioprotective and antifibrotic effects of sildenafil and riociguat have been demonstrated in previous studies [39], [40], [41]. Schymura et al. demonstrated that the sGC stimulator BAY 41–8543 increased RV contractility and reduced fibrosis in a pulmonary artery banding model of RVH [42]. In our study, we demonstrated that both compounds prevented further development of occlusive lesions and progression of disease in SUHx rats. However, the invasive and noninvasive measurements of hemodynamics and heart function demonstrated that RV function significantly improved in response to sildenafil and riociguat, compared with pre-treatment and SUHx 3-week control values. These findings could indicate a dual effect of both compounds on pulmonary vasculature and heart function. Nonetheless, further study of the effects of riociguat on RVH and RV function in a pulmonary artery banding model of RVH is warranted.

To our knowledge, this study is the first to describe the successful therapeutic use of the sGC stimulator riociguat in a well-accepted animal model of severe PAH and RVH. We have demonstrated that riociguat effectively suppresses lung vascular remodeling and significantly improves RV function, as measured via a range of invasive and noninvasive cardiopulmonary endpoints.

## Supporting Information

Figure S1 **RV staining with picrosirius red to show collagen content in SUHx rats treated with sildenafil and riociguat.** Representative images showing the collagen content in different treatment groups.
(TIF)

Figure S2 **Correlation between RVSP and the proportion of occluded vessels.** The correlation coefficient between RVSP and the proportion of occluded vessels is r^2^  = 0.66, *p*<0.003.
(TIF)
