# Supplementary figures and images for: The Soluble Guanylate Cyclase Stimulator Riociguat Ameliorates Pulmonary Hypertension Induced by Hypoxia and SU5416 in Rats
Source: PLoS One. 2012 Aug 17;7(8):e43433. doi: 10.1371/journal.pone.0043433 (PMC3422306; doi:10.1371/journal.pone.0043433)

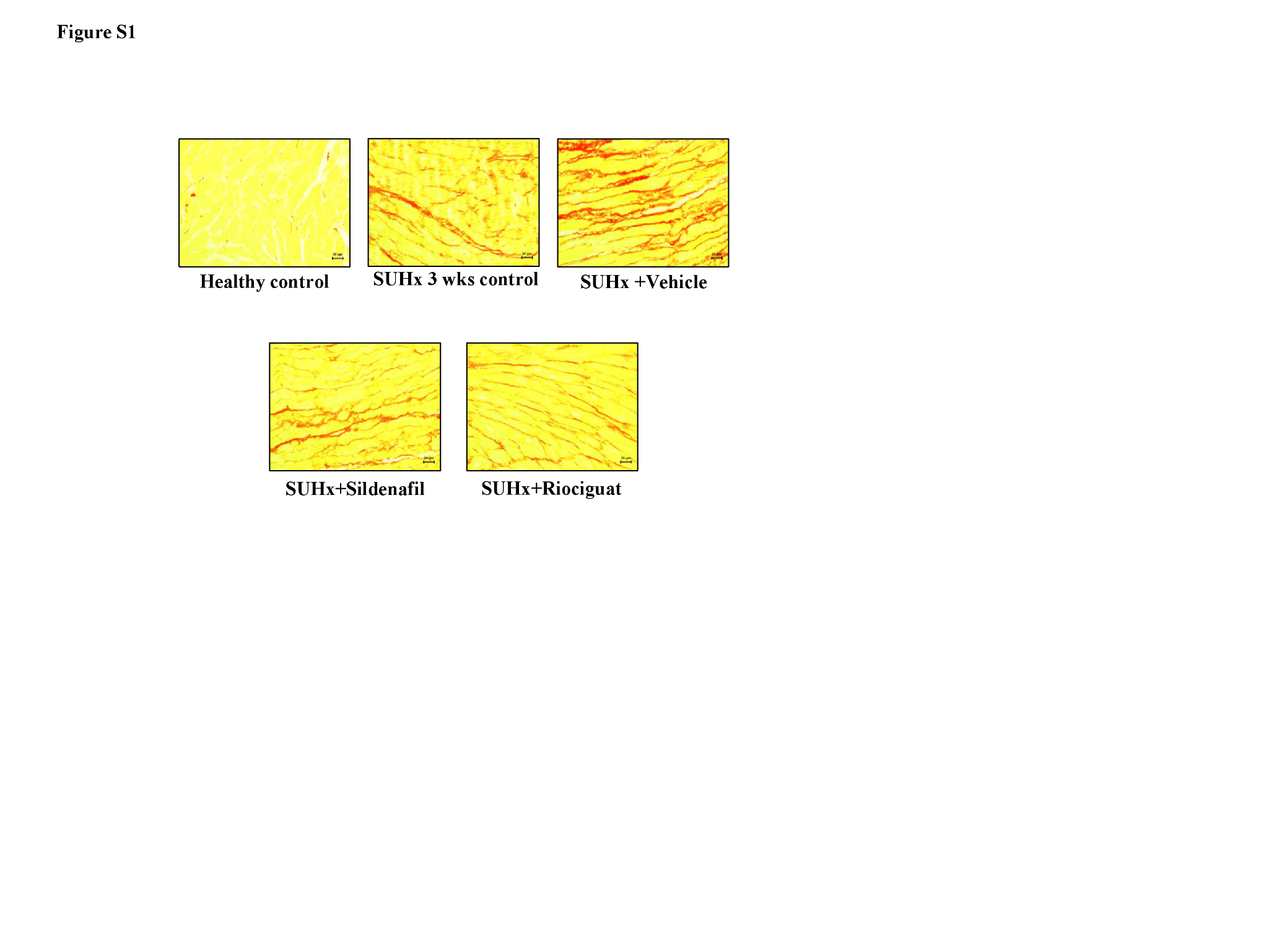

Supplement: Figure S1 — RV staining with picrosirius red to show collagen content in SUHx rats treated with sildenafil and riociguat. Representative images showing the collagen content in different treatment groups. (TIF) [file pone.0043433.s001.tif]

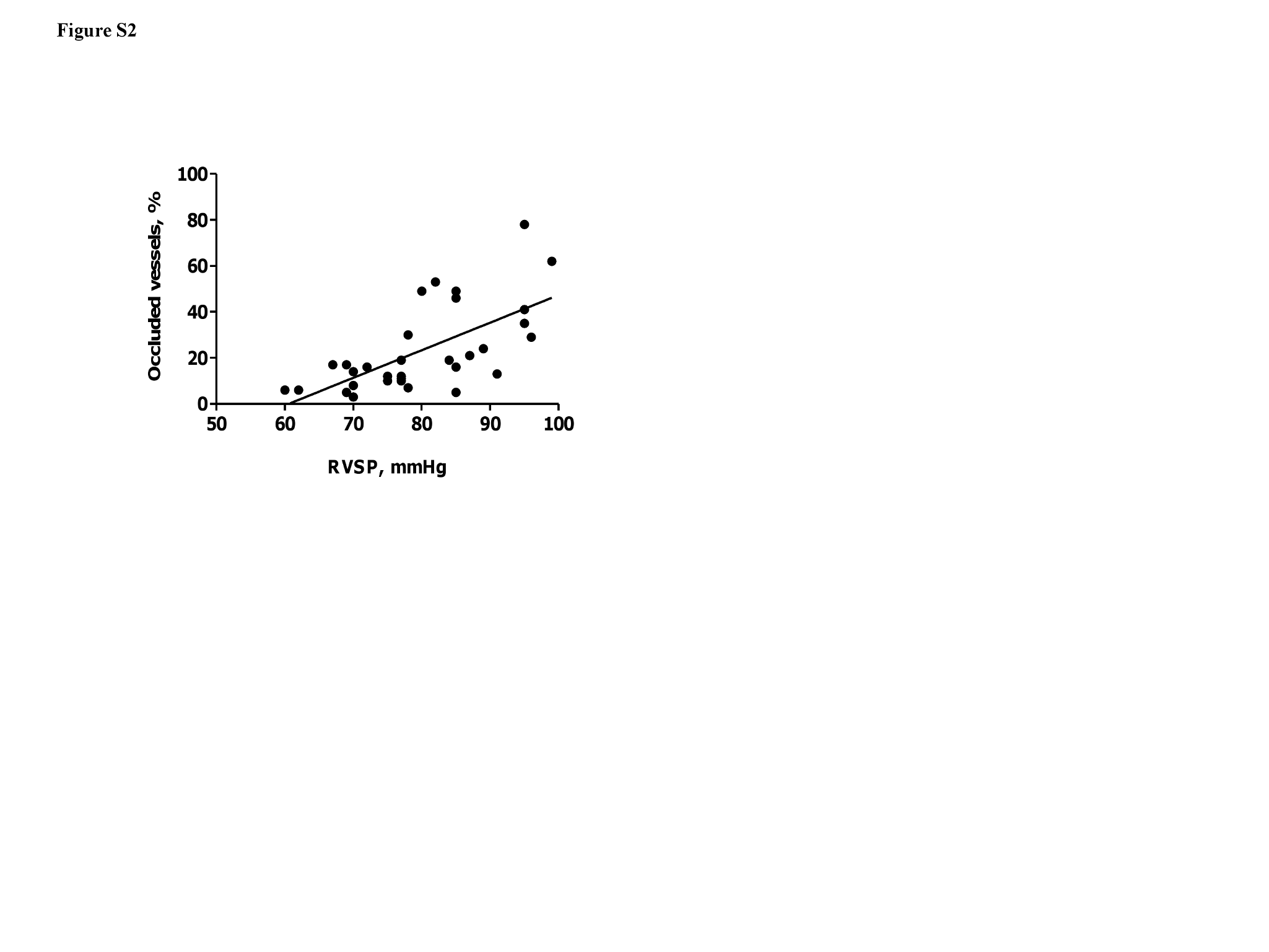

Supplement: Figure S2 — Correlation between RVSP and the proportion of occluded vessels. The correlation coefficient between RVSP and the proportion of occluded vessels is r2 = 0.66, p<0.003. (TIF) [file pone.0043433.s002.tif]
